# Pennsylvania Hospital—Surgical Wards—Service of Dr. Peace

**Published:** 1850-12

**Authors:** W. H. Gobrecht

**Affiliations:** Resident Surgeon


*Pennsylvania Hospital—Surgical Wards—Service of* DR. PEACE. *Cases discharged from April 1st to May 1st,* 1850.

| Disease. | Cured. | Average time under treatment in days. | By request. | Average time under treatment in days. | Died. | Average time under treatment in days | Tot. |
|---|---|---|---|---|---|---|---|
| Abscess - - | 1* | 41 | 0 | 0 | 1† | 15 | 2 |
| Burns - - | 3 | 41.3 | 1 | 1 | 0 | 0 | 4 |
| Caries - - | 1 | 212 | 0 | 0 | 0 | 0 | 1 |
| Conjunctivitis (chr) | 0 | 0 | 1 | 227 | 0 | 0 | 1 |
| Contusion - | 5 | 8 | 0 | 0 | 0 | 0 | 5 |
| Corneal opacity | 1 | 75 | 1 | 45 | 0 | 0 | 2 |
| Fistula in perin. | 1 | 320 | 0 | 0 | 0 | 0 | 1 |
| recto-vag. | 1 | 17 | 0 | 0 | 0 | 0 | 1 |
| Fractures, 15 simple 12, viz.: | | | | | | | |
| Humerus - | 1 | 39 | 0 | 0 | 0 | 0 | 1 |
| Radius - - | 1 | 32 | 0 | 0 | 0 | 0 | 1 |
| Sternum, - | 0 | 0 | 1 | 14 | 0 | 0 | 1 |
| Rib, - - | 2 | 27.5 | 0 | 0 | 0 | 0 | 2 |
| Spine - - | 0 | 0 | 0 | 0 | 1 | 1 | 1 |
| Pelvis, - - | 0 | 0 | 0 | 0 | 1 | 6 | 1 |
| Leg - - | 3 | 76.7 | 2 | 12 | 0 | 0 | 5 |
| Compound 3, viz.: | | | | | | | |
| Nose - - | 1 | 18 | 0 | 0 | 0 | 0 | 1 |
| Hand, - - | 0 | 0 | 0 | 0 | 1‡ | 67 | 1 |
| Foot, - - | 1 | 177 | 0 | 0 | 0 | 0 | 1 |
| Frost-bite - | 1 | 98 | 0 | 0 | 0 | 0 | 1 |
| Gangrene - | 1 | 85 | 0 | 0 | 0 | 0 | 1 |
| Gonorrhœa - | 1 | 24 | 0 | 0 | 0 | 0 | 1 |
| Hydrocele - | 0 | 0 | 1 | 3 | 0 | 0 | 1 |
| Inflammation of arm | 1 | 24 | 0 | 0 | 0 | 0 | 1 |
| hip | 0 | 0 | 1 | 136 | 0 | 0 | 1 |
| knee | 2 | 35 | 0 | 0 | 0 | 0 | 2 |
| leg | 1 | 107 | 0 | 0 | 0 | 0 | 1 |
| Iritis - - | 1 | 42 | 0 | 0 | 0 | 0 | 1 |
| Luxation - - | 1‖ | 26 | 1§ | 19 | 0 | 0 | 2 |
| Lupus - - | 0 | 0 | 1 | 2 | 0 | 0 | 1 |
| Necrosis - - | 1 | 335 | 0 | 0 | 0 | 0 | 1 |
| Paronychia - | 0 | 0 | 1 | 54 | 0 | 0 | 1 |
| Syphilis - - | 7 | 31.9 | 2 | 52.5 | 0 | 0 | 9 |
| Ulcer - - | 2 | 146.5 | 0 | 0 | 0 | 0 | 2 |
| Wounds 12, viz.: | | | | | | | |
| Gunshot - | 1 | 45 | 0 | 0 | 0 | 0 | 1 |
| Incised - - | 3 | 26.7 | 1 | 3 | 0 | 0 | 4 |
| Lacerated - | 3 | 56.7 | 0 | 0 | 1¶ | 9 | 4 |
| Punctured - | 3 | 38.7 | 0 | 0 | 0 | 0 | 3 |
| | 51 | | 14 | | 5 | | 70 |

* This was Mammary.

†This was situated at the upper extremity of the sternum, involving the sterno-clavicular articulation on the right side, and eventually communicated with the anterior mediastinum, assuming a gangrenous condition before death.

‡ The inflammation which here arose in a man of depraved constitution, extended throughout the arm, and the patient died from the exhaustion consequent upon the profuse purulent discharge, he being at no time, after the very proper attempt to save the fingers, in a suitable condition for amputation.

‖ Of clavicle. § Of humerus. ¶ Of scalp.

W. H. GOBRECHT, M. D., Resident Surgeon.

